# The role of mechanosensitive ion channels in the gastrointestinal tract

**DOI:** 10.3389/fphys.2022.904203

**Published:** 2022-08-19

**Authors:** Haoyu Yang, Chaofeng Hou, Weidong Xiao, Yuan Qiu

**Affiliations:** ^1^ Experimental Center of Basic Medicine, College of Basic Medical Sciences, Army Medical University, Chongqing, China; ^2^ Department of General Surgery, Xinqiao Hospital, Army Medical University, Chongqing, China

**Keywords:** mechanosensation, mechanotransduction, ion channels, mechanical stimuli, gastrointestinal disease

## Abstract

Mechanosensation is essential for normal gastrointestinal (GI) function, and abnormalities in mechanosensation are associated with GI disorders. There are several mechanosensitive ion channels in the GI tract, namely transient receptor potential (TRP) channels, Piezo channels, two-pore domain potassium (K2p) channels, voltage-gated ion channels, large-conductance Ca^2+^-activated K^+^ (BKCa) channels, and the cystic fibrosis transmembrane conductance regulator (CFTR). These channels are located in many mechanosensitive intestinal cell types, namely enterochromaffin (EC) cells, interstitial cells of Cajal (ICCs), smooth muscle cells (SMCs), and intrinsic and extrinsic enteric neurons. In these cells, mechanosensitive ion channels can alter transmembrane ion currents in response to mechanical forces, through a process known as mechanoelectrical coupling. Furthermore, mechanosensitive ion channels are often associated with a variety of GI tract disorders, including irritable bowel syndrome (IBS) and GI tumors. Mechanosensitive ion channels could therefore provide a new perspective for the treatment of GI diseases. This review aims to highlight recent research advances regarding the function of mechanosensitive ion channels in the GI tract. Moreover, it outlines the potential role of mechanosensitive ion channels in related diseases, while describing the current understanding of interactions between the GI tract and mechanosensitive ion channels.

## 1 Introduction

In healthy adult humans, the GI tract is responsible for the management of approximately 9–11 L of fluid and large amounts of solids and semisolids that pass through the intestinal lumen every day (Ghishan and Kiela, 2012). Besides secretion and absorption, the GI tract also senses mechanical stimuli to coordinate the digestion and absorption of nutrients and waste excretion. Furthermore, during digestion, the GI tract transfers signals regarding the composition of the intestinal contents to other organs so as to prepare the metabolic and cardiovascular systems for large amounts of absorbed nutrients. This process can also transfer the information of the required chemicals to other cells to regulate metabolic mechanisms (Le et al., 2021).

The intestinal mucosa is exposed to a variety of mechanical stimuli under normal and pathophysiological conditions. These mechanical stimuli include deformation, pressure, compression, and shear stress generated by peristaltic contractility, villous motility interaction with luminal contents, and mucosal remodeling and healing (Basson, 2003; Perez-Gonzalez et al., 2022). The bowel wall is exposed to constant tension, determined by its shape, mechanical characteristics, and muscular contraction (Basson, 2003). This tension modulates many important cell physiological functions, such as cell signaling, mechanosensation, and shape changes (Chao and Sachs, 2021).

Mechanical stimuli can be converted into biochemical signals by mechanosensitive cells, through a process termed mechanotransduction. Mechanotransduction is the process by which cells convert mechanical stimuli from their extracellular or intracellular forces into biochemical signals, which then trigger downstream cellular responses (Retailleau and Duprat, 2014). The ability to sense mechanical stimuli and achieve mechanotransduction, known as mechanosensitivity, is a crucial component of GI function (Joshi et al., 2021). In the GI tract, membrane deformation of the different mechanosensitive cells that form mechanoreceptors is generally hypothesized to induce the activation of mechanosensitive ion channels, which in turn modulate mechanotransduction. These mechanosensitive cells include intestinal epithelial cells, SMCs, and enteric neurons. Thus, the intestinal cells that form mechanoreceptors express ion channels that are activated by mechanical forces in the bowel lumen. In this way, they act as mechanodetectors and mechanotransducers (Del Valle et al., 2012).

In the GI tract, mechanosensitive cells sense mechanical forces and transduce them into electrical signals by mechanosensitive ion channels (Hamill and Martinac, 2001). Mechanotransduction consists of two processes. Firstly, mechanosensitive cells sense mechanical stimuli by using mechanoreceptors. Secondly, cells transduce receptor signals into their corresponding downstream responses using mechanotransducers (Joshi et al., 2021). Cells may use a series of mechanoreceptors, namely sarcomeric proteins, ion channels, cell surface receptors, and transmembrane adhesion receptors (Gayer and Basson, 2009; Liu et al., 2017; Pang et al., 2018; Wolfenson et al., 2019; Sheetz, 2021; Lin et al., 2022). Functionally, the intestinal layers have distinct mechanical characteristics and can sense a variety of mechanical forces, finally leading to appropriate GI function (Joshi et al., 2021). For example, EC cells in the GI epithelium are specialized mechanosensors that release 5-hydroxytryptamine (5-HT) in response to mechanical forces. Previous efforts to characterize intestinal mechanical stimuli have focused on the secretion of GI hormones by specific cells that sense said mechanical stimuli. However, recent research developments have revealed that many GI cell types can sense mechanical stimuli and perform their corresponding functions. In this review, we highlight recent advances in our understanding of the structure and function of mechanosensitive ion channels in the GI tract. We then summarize how mechanosensitive ion channels are located and function on various intestinal cells. Finally, we summarize existing knowledge regarding the potential association between mechanosensitive ion channels and intestinal diseases.

## 2 Mechanosensitive ion channels in the gastrointestinal tract

**FIGURE 1:**
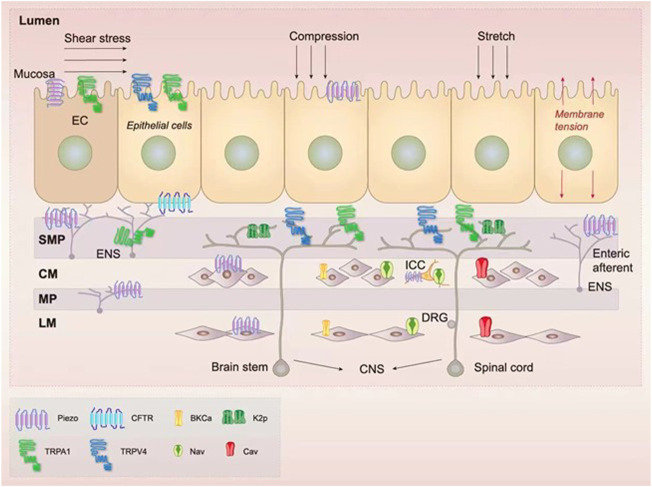
Expression of the mechanosensitive ion channels in the gastrointestinal (GI) tract. There are several mechanosensitive ion channels in the GI tract, namely transient receptor potential (TRP) (Brierley et al., 2008; Nozawa et al., 2009; Balemans et al., 2017; Alaimo and Rubert, 2019), Piezo (Bai et al., 2017; Mazzuoli-Weber et al., 2019), two-pore domain potassium (K2p) (Alcaino et al., 2017; Ma et al., 2018), voltage-gated ion channels (Farrugia et al., 1999; Strege et al., 2003b), large-conductance Ca^2+^-activated K^+^ (BKCa) channels (Ghatta et al., 2006; Singh et al., 2012), and the cystic fibrosis transmembrane conductance regulator (CFTR) (Gadsby et al., 2006). These channels are located in many mechanosensitive intestinal cell types, namely enterochromaffin (EC) epithelial cells (Wang et al., 2017), interstitial cells of Cajal (ICCs) (Ranade et al., 2015), smooth muscle cells (SMCs) (Joshi et al., 2021), intrinsic neurons [including myenteric plexus (MP) and submucosal plexus (SMP)] (Alcaino et al., 2017), and extrinsic enteric neurons [composed of neurons from the central nervous system (CNS)] (Alcaino et al., 2017). In the intestinal lumen, a variety of mechanical forces such as shear stress, compression, stretch, and membrane tension can be sensed by mechanosensitive ion channels (Hamill and Martinac, 2001; Alcaino et al., 2017).

Mechanosensitive ion channels are transmembrane proteins that are gated by mechanical forces that form ion currents. They can also be activated by other stimuli, such as heat or chemical stimuli (Blackshaw, 2014; Yu et al., 2016). In the GI tract, mechanosensitive ion channels consist of TRP channels, Piezo channels, K2p channels, voltage-gated Na^+^ (Nav) and Ca^2+^ (Cav) channels, BKCa channels, and the CFTR (Figure 1) (Zhang et al., 2010; Coste et al., 2012; Berrier et al., 2013; Gu and Gu, 2014; Mazzuoli-Weber and Schemann, 2015; Neshatian et al., 2015; Volkers et al., 2015; Yu et al., 2016; Alcaino et al., 2017).

### 2.1 Transient receptor potential channel families

The mammalian TRP superfamily consists of 28 TRP members, which are classified into six subgroups according to their amino acid sequence homology: TRPP, TRPC, TRPM, TRPML, TRPV, TRPN, and TRPA. Of these subgroups, TRPV4 and TRPA1 are the most closely associated with mechanical stimuli (Yu et al., 2016; Balemans et al., 2017; Startek et al., 2019; Karki and Tojkander, 2021). In the human colon, TRPV4 is situated in fine nerve fibers associated with blood vessels in the submucosa and serosa, while the myenteric plexus (MP) and the longitudinal and circular smooth muscles are mostly negative for its expression (Brierley et al., 2008). In the murine colon, TRPV4 is located in epithelial cells and mucosal glial cells of the muscular and submucosal layers (Alaimo and Rubert, 2019). Studies into murine models of embryonic explants have shown that TRPV4 can modulate cellular morphogenesis. Its activation regulates smooth muscle contractility, which generally supports organ development (Morgan et al., 2018). Luo et al. (2018) showed that TRPV4-responsive muscularis macrophages mediated contractility by directly interacting with SMCs, independent of neuronal input. TRPV4 can also integrate mechanical stimuli from different environments that are converted into Ca^2+^ signals, promoting various responses in different tissues (Garcia-Elias et al., 2014). Cui et al. (2021) found that for most Ca^2+^-dependent and secretagogue-induced intestinal anion secretion, the serosal TRPV4-constituted SOCE mechanism is likely universal. Fichna et al. (2015) demonstrated that TRPV4 channels are located in myenteric neurons in the mouse colon and play a significant inhibitory role in colonic motility regulation. Their study suggested that the mechanism of TRPV4-mediated relaxant action requires NO generation and intracellular and extracellular Ca^2+^. TRPV4 is also situated in primary spinal afferent neurons innervating the colon. The activation of protease-activated receptor-2(PAR2) increases currents in these neurons, prompts the discharge of action potentials from colonic afferent fibers, and induces mechanical hyperalgesia (Sipe et al., 2008). Besides TRPV4, TRPA1 also plays a crucial role in mechanosensation in the GI tract. In the mammalian GI tract, TRPA1 is situated in both extrinsic afferent neurons and intrinsic enteric neurons (Balemans et al., 2017). TPRA1 is also located in nonneuronal 5-HT-releasing EC cells (Nozawa et al., 2009), cholecystokinin-releasing endocrine cells (Purhonen et al., 2008), and other intestinal epithelial cells (Purhonen et al., 2008; Balemans et al., 2017; Alaimo and Rubert, 2019). TRPA1 is a non-selective, homotetrameric cation channel that is activated by a range of exogenous or endogenous compounds (Meents et al., 2019; Souza Monteiro de Araujo et al., 2020). TRPA1 is assumed to function as a mechanosensor and pain sensor in various human tissues and plays a vital role in detecting mechanical stimuli by visceral afferent fibers (Uckert et al., 2017; Moore et al., 2018; Giorgi et al., 2019). Brierley et al. (2009) found that TRPA1 mRNA expression is enriched within GI sensory neurons, whilst in the periphery, TRPA1 protein is localized within nerve endings at sites where mechanical stimuli are transduced. Under inflammation, TRPA1 appears to play an important role as a secondary transducer of many proinflammatory mediators, such as endogenous PAR-2 agonists, bradykinin, and capsaicin. PAR-2 (Cattaruzza et al., 2010) and Bradykinin (Balemans et al., 2017) functionally sensitize TRPA1 to increase mechanosensory function, which is absent in TRPA1 gene knock-out mice (Balemans et al., 2017), while capsaicin functionally desensitizes TRPA1 to decrease mechanosensory function (Brierley et al., 2009; Balemans et al., 2017; Moparthi and Zygmunt, 2020). TRPA1 is present in nociceptive neurons innervating the colon, where TRPA1 activation causes hypersensitivity to colorectal distention. TRPA1 mediates visceral hypersensitivity (VH) induced by PAR2 and trinitrobenzene sulfonic acid (TNBS, an inflammatory agent) (Cattaruzza et al., 2010). However, TRPA1 mechanosensitivity in hair cells has been questioned. TRPA1-deficient mice exhibit no vestibular defects and show normal auditory function, suggesting that TRPA1 is not required for hearing mechanotransduction (Bautista et al., 2006; Kwan et al., 2006). The activity of human TRPA1 (hTRPA1) is abolished by the thiol reducing agent TCEP (Moparthi and Zygmunt, 2020). The mechanosensitivity of hTRPA1 is dependent on its redox state, and it is suggested that oxidative stress transforms hTRPA1 into a protein conformation that is sensitive to mechanical stimuli (Moparthi and Zygmunt, 2020).

### 2.2 Piezo channels

In 2010, Coste et al. (2012) screened and first identified Piezo1, a mechanosensitive ion channel protein, in the glioma Neuro2A cell line. They subsequently found its subtype, Piezo2, through sequence homology (Saotome et al., 2018). Piezo1 and Piezo2 each have a unique propeller-like structure and are associated with mechanotransduction in various key processes (Fang et al., 2021). Piezo channels are expressed throughout the digestive system in humans and mice, such as in epithelia, the enteric nervous system, and SMCs (Bai et al., 2017; Mazzuoli-Weber et al., 2019). Specifically, the Piezo2 channel is highly and specifically expressed in human and mouse EC cells (Wang et al., 2017).

Piezo channels act in mechanotransduction pathways and other developmental signaling networks, such as proliferation and differentiation pathways. Piezo1 and Piezo2 interact with several critical mechanotransduction proteins such as transmembrane protein 150C (Anderson et al., 2018) and Stomatin like 3 (Qi et al., 2015). They are also associated with negative regulator proteins such as sarco/endoplasmic reticulum Ca^2+^-ATPase (Zhang et al., 2017) and Polysystin-2 (Peyronnet et al., 2013). Furthermore, Annexin A6 (ANXA6) regulates intracellular Ca^2+^ stores by negatively interacting with Piezo channels (Raouf et al., 2018). The Piezo channels regulate neurogenic locus notch homolog protein 1 (Notch1) signaling by activating the ADAM10 sheddase, playing a significant role in regeneration and cell differentiation pathways (Alabi et al., 2016). Notch1 is also associated with cell fate determination, by acting as a receptor for Delta1, Jagged1, and Jagged2 like ligands (Shimizu et al., 2000; Qin et al., 2019).

### 2.3 K2p channels

The K2p channel is a major and structurally distinct subgroup of the mammalian K^+^ channel superfamily (Enyedi and Czirjak, 2010). Three K2p channel subtypes (TRAAK, TREK-1, and TREK-2) exist in the extrinsic afferent neurons that innervate the mouse colon (Alcaino et al., 2017; Ma et al., 2018).

TREK and TRAAK are mechanically activated and can sense negative membrane pressure and shear stress (Cox et al., 2019; Lengyel et al., 2021). When negative pressure (such as hydrostatic pressure) is applied to a membrane, TREK and TRAAK produce a progressive, non-inactive current. Interestingly, the mechanosensitivity of individual cells was found to be retained in the patch-clamp technique (Cox et al., 2019; Lengyel et al., 2021). This suggests that TRAAK and TREK channels are mechanotransducers. Another study showed that TREK-1 was related to cell membrane polarization, volume regulation, and shear stress sensing formed by fluid flow through blood vessels (Noel et al., 2011). Taken together, these results suggest that TRAAK, TREK-1, and TREK-2 are mechanosensitive channels that might play a critical role in colon sensory damage through a disinhibitory mechanism (Kefauver et al., 2020).

### 2.4 Voltage-gated ion channels

Nav channels are complete membrane glycoproteins that consist of an α-subunit of 260 kDa in the center and several β-subunits of approximately 35 kDa (Dib-Hajj et al., 2009). In the GI tract, Nav channels are located in both the ICCs and SMCs of the circular smooth muscle layer of the human jejunum (Holm et al., 2002; Ou et al., 2002; Strege et al., 2003b), dog jejunum (Strege et al., 2007), and rat ileum (Smirnov et al., 1992) and gastric fundus (Smirnov et al., 1992), as well as in the SMCs of rat and human colons (Xiong et al., 1993; Alcaino et al., 2017; Campos-Rios et al., 2021). In the human jejunum and colon, shear stress activates Nav1.5 currents in a cytoskeletal-dependent manner; membrane lipids also play a significant role (Neshatian et al., 2015). However, there are species differences in the expression of the SCN5A-encoded Nav1.5 channel. SCN5A mRNA is expressed in the circular muscle of humans, mice, and dogs but not in that of pigs or guinea pigs . Strege et al. (2007) found that compared with wild-type, *SCN5A* mutation G615E Nav1.5 mice had decreased SMCs excitability, suggesting that Nav1.5 mechanosensitivity may play a crucial excitatory role in SMC mechano-electrical feedback (Strege et al., 2019). Furthermore, the Nav1.5 channel is inherently sensitive to membrane stretch: in a fully reversible manner, stretch evoked the acceleration of the rate-limiting voltage-dependent step leading to ionic current activation and inactivation (Morris and Juranka, 2007; Morris, 2011). The influx of Na^+^ through Nav1.5 produces a fast depolarization in membrane potential, which is crucial for electrical slow waves in GI SMCs (Strege et al., 2019). Nav1.6 is closely associated with mechanical pain-sensing and pain transmission in the lower organs (Israel et al., 2019). Specifically, this channel is found at the end of the pelvic tension-sensitive unmyelinated colon sensory afferent (Feng et al., 2015). Moreover, it is activated by colon stretch (Feng et al., 2015). Several experimental studies have demonstrated that Nav1.7 plays an important role in pain transduction, such as inflammation-related pain and mechanical pain (Nassar et al., 2004; Bi et al., 2017; Campos-Rios et al., 2021; Zhang et al., 2021).

Besides the Nav channel, the Cav channel is a crucial part of the GI tract. Cav channels are the key signaling sensors for electrical excitability; they transduce cell membrane action potentials to the downstream Ca^2+^ transients. Upon entering the cytosol, Ca^2+^ regulates enzyme activation and gene expression (Altmuller et al., 2017). Recent studies have found that L-Cav also contributes to the proliferation and differentiation of several kinds of stem cells (Tan et al., 2019). Cav channels are expressed in human GI SMCs (Farrugia et al., 1999) and are a significant factor regarding the membrane potential and excitability of SMCs. GI L-type Cav1.2 channels have also been shown to be sensitive to both mechanical osmotic and shear stress (Farrugia et al., 1999; Strege et al., 2003a; Kim et al., 2007; Anishkin et al., 2014).

### 2.5 Other channels

BKCa channels belong to a unique group of ion channels that are activated by both membrane depolarization and cytosolic Ca^2+^. This channel is characterized by its large conductance and senses intracellular Ca^2+^, hyperpolarizes the cell membrane, and controls the excitability and functions in various tissues (McManus and Magleby, 1991; Brenner et al., 2000). The stress-regulated exon insert is an indispensable domain for the mechanosensitivity of BKCa (Wang et al., 2010). The BKCa channel is a key channel in colonic smooth muscle. BKCa channels also provide a large outward current to the smooth muscles to decrease excitability (Ghatta et al., 2006; Singh et al., 2012).

CFTR is an anion and intracellular ligand-gated channel related to cystic fibrosis (CF) (Gadsby and Nairn, 1999). CFTR is situated in the apical membrane in various epithelial tissues of the human body, including the GI tract and pancreatic ducts (Gadsby et al., 2006). CFTR is a key determinant of ion and water homeostasis (Saint-Criq and Gray, 2017). CFTR is highly expressed at the base of the crypt, playing an important role in influencing the intestinal stem cell compartment (Barker et al., 2007; Jakab et al., 2011; Anderson et al., 2019). CFTR could modulate volume decrease in epithelial cells directly by responding to hypotonicity-induced membrane stress (Vitzthum et al., 2015; Xie et al., 2016).

## 3 Mechanosensitive cells in the gastrointestinal tract

### 3.1 Mechanosensitivity of gastrointestinal epithelium

The integrity and dynamic balance of the epithelium are essential for survival. They also allow the epithelium to form an intelligent dynamic barrier between the external environment and internal organs (Macara et al., 2014). As with other epithelia, such as those of the bladder and the kidney, the GI epithelium is mechanosensitive. Moreover, it encounters multiple mechanical forces, ranging from smooth muscle contraction to shear stress (Beyder, 2018). Intestinal epithelial cells, which include Paneth cells, goblet cells, endocrine cells, and absorptive epithelial cells, are all mechanosensitive (Basson, 2003; Gayer and Basson, 2009; Shaw et al., 2012). The mechanosensitivity of these intestinal epithelial cells has long been a concern about how various forces interact with the digestive, absorption, secretion, and immune function of the GI tract.

EC cells are the most numerous neuroendocrine epithelial cells that line the lumen of the GI tract (Martin et al., 2018). EC cells have long been a topic of considerable research regarding mechanosensitive endocrine cells. By acting as “mechanical sensors,” EC cells function as sensory detectors to detect mechanical forces during intestinal peristalsis. EC cells can sense a range of mechanical forces, including flow shear stress, stretch/distension, membrane distortion/deformation, tensile force, touch, intraluminal pressure, compression, turbulent and centrifugal forces, and cell volume changes (Linan-Rico et al., 2016; Beyder, 2018). These mechanical forces result in the release of 5-HT, which promotes or activates intestinal neural reflex, the coordination of numerous movements, secretion, mixed movements (fed state), and stool reflexes (Linan-Rico et al., 2016). Studies have shown that purines, particularly ATP, play a central role in mechanically stimulating 5-HT release in EC cells (Maffei, 2020). Nevertheless, it remains unclear as to whether these receptors act as major mechanical receptors. Wang et al. (2017) found that Piezo2 messenger ribonucleic acid (mRNA) was located in both murine and human small intestinal epithelial cells. Piezo2 immunolabeling, meanwhile, has revealed a specific distribution of these channels in human jejunum 5-HT positive EC cells. Piezo2 channels serve as the mechanical sensor in EC cells. Activated by mechanical force, Piezo2 facilitates 5-HT release and increases mucosal secretion, such as chloride secretion (Wang et al., 2017). However, it remains unknown whether other ion channels such as K2p channels and Ca^2+^ channels modulate 5-HT release.

Regarding epithelial cells, here this review focuses on two functions that mechanical stimuli cause in the epithelium. Firstly, mechanical stimuli promote the proliferation, differentiation, and repair of the intestinal epithelium (Shaw et al., 2012). Enteric epithelial cells are prone to repeating deformation during peristalsis and villus movement. However, under abnormal stimulation, such as sepsis or intestinal obstruction, the mucosa shrinks. This repeated deformation stimulates the proliferation of intestinal epithelial cells, through both focal adhesion kinase (FAK) and extracellular signal-regulatory kinase (ERK) (Chaturvedi et al., 2007). The intestinal mucosa is in constant contact with pathogens, toxins, and allergens, so it must be repaired to maintain the mucosal barrier (Suzuki, 2020). Previous *in vivo* studies have shown that mechanical forces, such as partial intestinal obstruction, regulate wound closure. *In vitro* studies have also demonstrated that the repeated deformation of intestinal monolayer epithelial cells stimulates Caco-2 or IEC-6 intestinal epithelial cells to close wounds in an ERK-dependent manner (on a fibronectin substrate) (Zeng et al., 2017). Secondly, normal epithelial cells can respond to mechanical forces. The piezo channel could shape stem cell zones by fission, which contributes to the GI epithelium self-renewing (Tallapragada et al., 2021). Malignant GI epithelial cells can also act in this manner, most obviously with increased cell adhesion, which is essential for tumor metastasis. For example, over-proliferating adjacent crypts lead to Notch overactivation. Furthermore, mechanical pressure caused by external forces has been shown to be related to enhanced β-catenin and Ret signing, resulting in abnormal crypt foci (Fernandez-Sanchez et al., 2015). Crypt fission occurs physiologically and complements crypt hyperplasia as a form of intestinal growth, but it is abnormally accelerated in colorectal adenomas and carcinomas (Tallapragada et al., 2021). Recent studies have demonstrated that, under physiological conditions, the Piezo1 channel senses both mechanical crowding and stretch. It may function as a homeostatic sensor to control epithelial cell numbers, causing cell division in sparse regions and apoptosis and extrusion in crowded regions (Gudipaty et al., 2017). The squeezing of live cells due to overcrowding from proliferation and migration helps to control the number of epithelial cells. Squeezing can be prevented by using blockers to destroy Piezo1 channels or achieving gene knockout; the formation of an epithelial cell mass can also be induced in this way. This cell mass formation can promote tumor growth, suggesting that Piezo1 plays a significant role in inhibiting epithelial tumors by providing a bridge of mechanical forces regarding the activation of intracellular biochemical pathways (Slattum and Rosenblatt, 2014).

### 3.2 Mechanosensitivity of gastrointestinal neurons

The enteric nervous system is composed of extrinsic and intrinsic neurons, which are pivotal for GI autonomic function. Intrinsic neurons are situated entirely within the GI tract, where they regulate GI peristalsis and secretion functions. The Soma of extrinsic neurons is contained outside the GI tract. Extrinsic neurons regulate GI peristalsis and modulate GI tract sensory function. Extrinsic and intrinsic neurons are both mechanosensitive.

#### 3.2.1 Mechanosensitivity of intrinsic enteric neurons

The MP and submucosal plexus (SMP) make up the GI intrinsic neurons (Phillips and Powley, 2007). The MP, which is the outer of the two enteric nervous plexuses, comprises a network of neurons that are located between the outer longitudinal muscle (LM) and circular muscle (CM) layers in the GI tract (Smith et al., 2007; Schemann and Mazzuoli, 2010). This plexus mainly contributes to the control of SMC motor patterns (Phillips and Powley, 2007). The SMP is situated in the submucosa, between the tunica muscularis and mucosa layers (Phillips and Powley, 2007). Most of the identified intrinsic neurons are directly mechanosensitive and act as both primary afferents and interneurons (Smith et al., 2007; Schemann and Mazzuoli, 2010; Alcaino et al., 2017; Mazzuoli-Weber et al., 2019). Unlike extrinsic spinal cord and vagal mechanoreceptors, whether their stimulation is sufficient seems to depend upon the length of the strain, rather than intramural tension (Hibberd et al., 2012). Experimental studies have shown that intestinal neurons have significant mechanosensitive responses to compression, shear stress, stretching, and hypoosmotic solutions (Hibberd et al., 2012; Dong et al., 2015; Kugler et al., 2015; Kugler et al., 2018; Filzmayer et al., 2020).

Dogiel type II neurons were once thought to be the only intrinsic sensory neurons in the enteric nervous system. They are activated by mechanical stimuli from the mucosa, which is dependent on 5-HT release from EC cells. Dogiel type II neurons can also respond to both contractions and stretching, with a constant action potential. Studies have shown that the expansion of the intestinal wall creates a reflex that is mediated by neurons, known as the peristaltic reflex (Brehmer, 2021). This reflex includes spatially coordinated muscle contractions and relaxations, which contribute to the passage of luminal contents. It is reasonable that the neuromechanosensors located in the MP are embedded between two layers of muscle, being therefore strategically localized to control movement and perceive deformations (Mazzuoli and Schemann, 2009). When expansion-induced contractile activity is impaired in the intestinal segment, myenteric neurons which are detached from the MP encode mechanical stimuli in order to trigger the peristaltic reflex. In the ileal intermuscular plexus of guinea pigs, rapidly adapted mechanosensitive enteric nerve cells have been demonstrated to trigger a phase-pulse discharge to respond to dynamic changes during intestinal deformation. This response has been shown to be reproducible and increase with force (Mazzuoli and Schemann, 2009). Rapid deformation can mimic changes in contractile tissue better than continuous stretching, thereby producing effective forces on neurons.

#### 3.2.2 Mechanosensitivity of extrinsic enteric neurons

The extrinsic innervation of the GI tract is provided by neurons from the central nervous system (CNS) that are situated in the brainstem, and in peripheral afferent ganglia (Mercado-Perez and Beyder, 2022). Extrinsic neurons do not play a key role in sensing intraluminal mechanical stimuli, so their mechanosensitivity is only discussed briefly here.

Spinal cord afferents have high expansion detection thresholds; they respond to a variety of stimuli extending to a wide range of injuries (Mercado-Perez and Beyder, 2022). Their cell bodies are located in the dorsal root ganglia (DRG), whose peripheral protrusions extend to the intestinal ganglia, mucosal epithelium, and muscle layer (Kim et al., 2022).

Studies have shown that vagal sensory neurons are mechanosensitive, with neuronal deformation regulating reflex activity (Berthoud et al., 2004). Vagal sensory neurons detect tension at a physiological level, which is essential for normal GI function (Andrews, 1986). The mechanoreceptors of sensory neurons in the vagus nerve can be divided into two categories: those that respond to the dilation and contraction of the intestinal wall, and those that can be triggered by the mechanical stimuli of the intestinal lumen (Page et al., 2002).

It is worth mentioning that the properties of extrinsic enteric neurons differ. First, compared with pelvic muscular afferents, splanchnic muscular afferents might respond to probing at lower stimulus intensities. Second, compared with splanchnic muscular afferents, pelvic muscular afferents exhibited greater responses to both stretch and probing. Third, the number of stretch-sensitive pelvic afferents is greater than stretch-sensitive splanchnic afferents. Taken together, the pelvic muscular afferents are better equipped than lumbar splanchnic afferents to respond to a colonic wall stretch (Brierley et al., 2004).

### 3.3 Mechanosensitivity of gastrointestinal smooth muscle cells

SMCs play a pivotal role in excitation-contraction coupling to transduce the electrical signal into contraction by voltage-gated channels (Joshi et al., 2021), which are key factors regulating the potential and excitability of SMC membranes. Na^+^ entry can be modulated by shear stress, which may change the contractile activity of intestinal SMCs. Blocking the intestinal SMC Na^+^ channel has been shown to lead to membrane hyperpolarization. Furthermore, with increasing shear stress, increased Na^+^ channels may depolarize the intestinal SMC, bringing the membrane potential closer to this contraction threshold (Joshi et al., 2021).

Farrugia et al. (1999) found that a stretch-activated, nifedipine-sensitive calcium channel exists in human jejunal circular SMCs. The channel is activated by external shear forces and increases in intracellular pressure. The presence of stretch-activated calcium channels in the GI SMCs may enable the SMCs to act as mechanotransducers and to function in the regulation of smooth muscle tone and intestinal motility.

Stretch-dependent K^+^ (SDK) channels are expressed in dog and mouse colonic SMCs. Stretch may regulate these channels by involving cytoskeletal interactions. SDK channels may play a significant physiologically role in maintaining membrane potential during cell elongation (e.g., during organ filling) and participating in intestinal inhibitory neural responses mediated by NO *via* cGMP-dependent pathways (Koh and Sanders, 2001).

The Piezo channel constitutes a further mechanosensitive ion channel in SMCs. Recent evidence shows that Piezo1 facilitates SMC development by regulating vascular endothelium shear stress sensitivity (Douguet et al., 2019). Moreover, it regulates the secretion of crosslinking enzymes, which are required to promote SMC remodeling (Retailleau et al., 2015).

The K2p channel appears to contribute to the regulation of colon motility, as its activation by agonists can effectively relax colon SMC tension (Ma et al., 2018). The TREK channel may offer a mechanism to counter stretch-activated currents, leading to depolarization and contraction. Hyperpolarization induced by stretching may constrict the response of contraction to distension. The outward rectification of TREK-1 increases the outflow of K2p at the depolarization voltage, thereby assisting repolarization. Thus, the activation of TREK-1 may increase the ability of cells to repeatedly produce depolarized electric activity, such as slow waves (Kraichely and Farrugia, 2007).

### 3.4 Mechanosensitivity of interstitial cells of cajals

Spanish Nobel Laureate physician Santiago Ramon y Cajal first described cells that are located between SMCs and the nerve endings in the GI tract (Foong et al., 2020). Currently, they are known as ICCs and belong to the SMC family, whose development requires kit signaling activation. Compared with SMCs, ICCs have fewer contractile elements but contain more mitochondria and endoplasmic reticulum. An ICC consists of a spindle cell body with a thin cytoplasm, a large oval nucleus, and dendritic processes (Sanders, 1996). ICCs have four important functions in the GI tract (Yin and Chen, 2008). Firstly, ICCs generate electrical slow wave activity. Secondly, they coordinate the active propagation of slow wave and pacemaker activity. Thirdly, ICCs transduce motor neural inputs from the enteric nervous system. Fourthly, ICCs are associated with myogenic stretch responses (Yin and Chen, 2008; Blair et al., 2014). For example, stretching of the mouse stomach and human jejunum has been shown to increase the frequency of slow waves, regardless of neuronal mechanisms (Treichel et al., 2018). Stretching of gastric antral muscles by approximately 25% with precise length ramps caused depolarization and increased slow wave frequency by non-neural mechanisms. The response to stretch is absent in muscles of W/W^v^ mice (which lack ICCs), suggesting that ICCs provide stretch sensitivity in gastric muscles. Products of arachidonic acid metabolism, such as prostaglandin E2, may mediate stretch-dependent responses, and the cyclooxygenase enzyme II, which is expressed in ICCs, may mediate the stretch sensor mechanism associated with the ICC (Won et al., 2005). Recent studies have shown that Na^+^ channels are expressed in human ICCs. Activated by shear stress, this native Na^+^ current in ICCs can modulate membrane potential and SMC contractile activity (Ranade et al., 2015).

## 4 Mechanosensitive ion channels and gastrointestinal disease

### 4.1 Mechanosensitive ion channels and irritable bowel syndrome

Rome IV defined IBS as a functional bowel disorder in which recurrent abdominal pain is associated with altered bowel habits. Disordered bowel habits are typically present (i.e., constipation, diarrhea, or a mix of constipation and diarrhea), as are symptoms of abdominal bloating/distension (Lacy and Patel, 2017). Ritchie et al. (1973) first found the pain from distension of the pelvic colon by inflating a balloon in IBS. The pain may be the outcome of a low visceral pain threshold for the part of the bowel in contact with the balloon. The altered intestinal motility in IBS patients is due to the abnormal mechanical stimuli detected by the mechanosensitive channel. In IBS patients, repeated distention of the distal sigmoid colon below the sensory threshold caused orad exaggerated motility of the colon. The distention inhibited small intestine motility in healthy subjects, but this inhibition was attenuated in IBS patients. These results show that IBS patients may have VH and an abnormal intestinal reflex (Fukudo et al., 2002). The secretion of chloride is important because it is the driving force for fluid movement into the intestinal lumen. Besides abnormal motility, intestinal secretion is also dysregulated. Mechanical distension causes reflexes on intrinsic afferent neurons, which triggers the dysregulation of 5-HT release and chloride secretion of EC Cells. This may be one mechanism of IBS (Xue et al., 2007).

In addition to motility and secretion abnormality, VH has become a crucial hypothesis for explaining the pain symptoms of IBS. Furthermore, it has been considered a biological marker of IBS (Fuentes and Christianson, 2016). Colorectal visceral pain is usually caused by cell swelling, instead of heating, extrusion, or punctures (Feng and Guo, 2020). Thus, ion channel mechanotransduction is crucial to the underlying mechanisms of IBS-related visceral pain. Ion channels such as Piezo, TRPV4, TRPA1, and Nav channels are thought to be the main mediators of VH (Sipe et al., 2008). This review summarizes the role of mechanosensitive ion channels in IBS.

Firstly, the expression of Piezo2 in the colon has been shown to be higher than that in the small intestine. Moreover, the visceral sensory threshold has been found to be negatively correlated with the expression of Piezo2 (Bai et al., 2017). These findings show that Piezo2 may be a potential VH biomarker. 5-HT plays a pivotal role in the visceral sensitivity of IBS, and IBS patients experience nociceptive responses that are induced by 5-HT (Keszthelyi et al., 2015). Piezo2 affects visceral nociception by modulating the release of 5-HT (Bai et al., 2017). Yang et al. (2016) injected Piezo2 short hairpin RNA (shRNA) into the sheath to reduce the expression of Piezo2 in the lumbar DRG. They revealed that control group rats showed significantly increased nociceptive sensation to non-noxious stimuli, while injected rats exhibited significantly reduced visceral sensation with both noxious and non-noxious stimuli (Yang et al., 2016). These findings suggest that Piezo2 inhibitors could be effective drugs for treating IBS.

Secondly, studies have shown that TRPV4 may be associated with pain and hyperalgesia (Balemans et al., 2017). Within this framework, biopsies of patients with IBS showed a significant increase in their number of nerve fibers expressing TRPV4 in the colon, which was correlated with their pain severity. Moreover, the expression of TRPV4 was found to be increased in preclinical models of VH (Jones et al., 2005; Fuentes and Christianson, 2016). TRPV4 is expressed by primary spinal afferent neurons innervating the colon. PAR2 activation increases currents in these neurons, evokes discharge of action potentials from colonic afferent fibers, and induces mechanical hyperalgesia. Furthermore, TRPV4 antagonists have been shown to reduce sensitivity to colorectal pain, and sensitivity to colorectal pain also decreases in TRPV4-knockout mice (Winston et al., 2007). Studies have shown that the upregulation and sensitization of TRPV4, which cause hyperalgesia, are partially affected by endocrine and inflammatory factors (Blackshaw et al., 2010). Taken together, these studies demonstrate the interconnected role of TRPV4 channels in the formation and maintenance of VH in IBS. The level of TRPV4 agonist 5,6-EET has been shown to be increased in biopsy samples from IBS patients. Therefore, the properties of over-generated TPRV4 channel agonists in IBS disease may represent new therapeutic opportunities to reduce channel activation (Cenac et al., 2015). Additionally, TRPV1 is also involved in VH in the GI tract and IBS pathogenesis (Blackshaw et al., 2010). Their activation causes pain, but it is followed by desensitization, which in turn results in analgesia. TRPV1 fast-desensitizing compounds may be promising agents in the treatment of IBS. One example is palvanil, which regulates intestinal motility and reduces visceral pain (Szymaszkiewicz et al., 2020).

Thirdly, under both normal and pathological conditions, the Nav1.1, Nav1.3, and Nav1.6 to Nav1.9 channels are related to nociceptor function (de Carvalho Rocha et al., 2014). However, Nav1.7 and Nav1.8 are more likely to play a role in VH regulation than other channels. Nav1.7 immunoreactive nerve fibers have been found to be higher in biopsy samples from patients with idiopathic rectal hypersensitivity than in those from healthy controls (Yiangou et al., 2007). Qu et al. demonstrated that neonatal colonic inflammation caused a significant increase in Nav channel activity in colon-specific DRG neurons in visceral hypersensitive adult rats. This activity was manifested by the upregulated expression of Nav1.7 and Nav1.8, and by an enhancement of the total Nav channel current density. In an *in vivo* rat study, blocking Nav1.8 alleviated mechanical hyperalgesia in the visceral cells of inflammatory pain models (Jarvis et al., 2007). In an experimental study of patients with IBS, the intra-rectal administration of lidocaine, which blocks Nav channels, was found to decrease abdominal pain and visceral hyperalgesia, highlighting a promising treatment target for IBS patients (de Carvalho Rocha et al., 2014).

Further studies into the exact mechanism of VH are needed. Moreover, a better understanding of the homeostatic and pathophysiological functions of VH will surely provide promising prospects for new IBS treatments.

### 4.2 Mechanosensitive ion channels and gastrointestinal tumors

In GI tumors, tumor tissues tend to be stiffer than the adjacent normal tissues. Tumor cells within a dilated tumor are exposed to intense mechanical forces, due to the increased extracellular matrix stiffness. Such forces could facilitate their malignant progression through mechanosensitive ion channels (Chin et al., 2016; Chaudhuri et al., 2018; Mohammadi and Sahai, 2018). Therefore, we mainly discuss the relationship between mechanosensitive ion channels and GI tumors.

By serving as a proto-oncogene, Piezo1 plays a crucial role in the development of many tumors in the GI tract. A germ-line dysfunctional mutation of Piezo1 has been found in some patients with colorectal adenomatous polyps (Spier et al., 2016). Immunohistochemical analysis of samples from patients with colon cancer revealed that Piezo1 expression was upregulated in cancer colon tissues and cells, compared with that in adjoining normal tissues (Sun et al., 2020). High levels of Piezo1 can reduce the survival rate of colon cancer patients. Furthermore, the upregulation of Piezo1 expression has been shown to facilitate the invasion and proliferation of colorectal cancer cells (Yu and Liao, 2021). Moreover, an *in vitro* study revealed that Piezo1 gene knockdown can reduce the invasion of colorectal cancer cells, highlighting the regulatory function of Piezo1 in colorectal cancer. Furthermore, these findings suggest potential biomarkers and therapeutic targets for colorectal cancer treatment (Sun et al., 2020).

K2p channels also play a pivotal role in regulating cell behavior associated with tumor development, including cell proliferation and migration. The expression of kv11.1 is altered in the K2p family, which has been shown to affect cell proliferation and apoptosis in colorectal cancer (Ousingsawat et al., 2007; Rodriguez-Rasgado et al., 2012). Potassium voltage-gated channel subfamily Q member 1 (KCNQ1) has been found to be very prominent in the K2p channels that lead to increased cancer risk (Liin et al., 2015). Moreover, KCNQ1 has been shown to serve as a tumor suppressor, as *kcnq1* mutant mice develop increased intestinal tumors, with some tumors progressing to become invasive adenocarcinomas (Starr et al., 2009; Than et al., 2014). In human colorectal cancer cells that were transferred to the liver, low KCNQ1 protein expression was found to be significantly associated with lower survival, compared with that in patients who demonstrated high KCNQ1 expression, whose survival was approximately 2 years longer (Than et al., 2014). Therefore, understanding the role of K2p channels in tumor development could reveal new perspectives regarding tumor treatments.

Liu et al. (2019) showed that TRPV4 expression was highly upregulated in colon cancer and related to poor prognosis. Inhibition of TRPV4 suppressed the growth of colon cancer cells by blocking the cell cycle in the G1 phase and inducing apoptosis and autophagic cell death (Liu et al., 2019). Matsumoto et al. (2020) demonstrated that TRPV4 in both macrophages and endothelial cells was involved in the regulation of AOM/DSS-induced colon carcinogenesis in mice. TRPV4 deficiency significantly reduced colitis-associated tumorigenesis and improved survival rate, compared with that in WT (Ou-Yang et al., 2018).

CFTR is a tumor suppressor in CRC. Endoscopic screening studies of adult CF patients showed that polyps in CF patients are larger and more aggressive than those in the non-CF population (Billings et al., 2014; Niccum et al., 2016). As a result of these studies, the Cystic Fibrosis Foundation has revised the endoscopic screening guidelines for patients with CF and has declared CF a hereditary colon cancer syndrome (Hadjiliadis et al., 2018). In addition, CFTR deficiency is associated with colorectal cancer. In an experiment including 90 patients with colorectal cancer, the disease-free survival at 3 years in the 25% of patients with the lowest CFTR expression was 30% lower than that in patients with a higher CFTR expression (Than et al., 2016).

## 5 Conclusion

Mechanosensitivity is essential for normal GI function. A variety of cell types in the GI wall are mechanosensitive, ranging from epithelial cells to both extrinsic and intrinsic neurons. These cells can transduce mechanical forces into biological signals through mechanosensitive ion channels. Both mechanosensitive voltage-gated ion channels and mechano-gated ion channels are essential for the fundamental function of the GI tract. Changes associated with mechanical stimuli accompanying GI inflammation and hypersensitivity, as well as the emergence of diseases related to mechanosensitive channel, have aroused great interest in the applicability of these molecular entities regarding treatments. Therefore, mechanosensitive ion channels can be viewed as a new strategy for drug intervention in GI diseases. Research regarding the relationship between intrinsic enteric neurons and mechanosensitive ion channels is limited, so further exploration is required. It remains puzzling, for example, as to how mechanical stimuli indirectly activate mechanosensitive ion channels. Therefore, much research is required to fully reveal the relevant mechanism of action. Nevertheless, as stated in this review, we believe that recent developments in this field mean that such applicability may become possible in the near future.
